# The Effects of Carum Carvi (Bunium Persicum Boiss) on Early Return of Bowel Motility After Caesarean Section: Double-Blind, Randomized, Placebo-Controlled Trial 

**Published:** 2019-03

**Authors:** Seyde Sedighe Yousefi, Omid Sadeghpour, Zeinab Hamzehgardeshi, Farnaz Sohrabvand

**Affiliations:** 1 Faculty of Medicine, Mazandaran University of Medical Sciences, Sari, Iran; 2 Traditional and Complementary Medicine Research Center, Mazandaran University of Medical Sciences, Sari, Iran; 3 The Research Institute for Islamic & Complementary Medicine, Iran University of Medical Sciences, Tehran, Iran; 4 Sexual and Reproductive Research Centre, Mazandaran University of Medical Sciences, Sari, Iran; 5 Department of Obstetrics, Gynecology & Infertility, School of Medicine, Tehran University of Medicine Sciences, Tehran, Iran

**Keywords:** Ileus, Caesarean Section, Bunium Persicum Boiss, Traditional Persian Medicine

## Abstract

**Objective:** To investigate the effect of the Carum carvi (Bunium persicum Boiss) plant, a gas solvent, on resumption of bowel motility after caesarean section.

**Materials and methods:** A randomized controlled trial was done on a group of 98 women undergoing elective caesarean section under general anesthesia in a university hospital. Patients in the intervention group (Group A) drank 10 ml of a syrup containing 8 g of Bunium persicum Boiss in 20 ml of syrup 6 to 7 hours after surgery. The control group (Group B) comprised 10 patients who drank 10 ml of placebo syrup 6 to 7 hours after surgery. Demographic characteristics, time to first hearing of normal intestinal sounds, time to first flatus, time to first bowel movement, and length of hospital stay were compared between the two groups.

**Results:** Compared with the control group, the intervention group had a significantly shorter mean time to hearing the first intestinal sounds (10.66 ± 2.38 vs. 19.54 ± 3.85 h), mean time to first flatus (13.91 ± 3.73 vs. 24.82 ± 5.83 h), mean time to first bowel movement (19.31 ± 4.63 vs. 30.70 ± 10.21 h), and mean length of hospitalization (31.70 ± 7.70 vs. 49.20 ± 10.16 h) (p < 0.05). No patients developed serious side effects associated with consumption of the syrup.

**Conclusion:** The use of a gas solvent such as Bunium persicum Boiss after caesarean section can speed the resumption of postoperative bowel motility.

## Introduction

Caesarean section is one of the most common surgical procedures performed in women. Recent studies in Iran indicate that 45.5% of women give birth via caesarean section (1). One side effect of this delivery method is decreased or delayed return of bowel motility after surgery, which is a problem that has not been addressed sufficiently (2). Typically, obstetricians do not introduce oral intake of food to the patient after caesarean section until bowel function returns. Return of bowel function is defined as bowel motility, passing gas, bowel movement, and feelings of hunger (3). 

Delaying the resumption of oral food intake can negatively affect production of the mother’s milk and breastfeeding and requires intravenous nutrition, which lengthens the hospital stay and increases the cost of postoperative care. It also increases the rate of cellular breakdown, delays healing, and increases the likelihood of infection (4).

The delay in resumption of oral intake of food stems from the belief that early oral intake can worsen postsurgical ileus (5). Ileus is a common and unpreventable consequence of abdominal surgery that is not life-threatening but significantly increases morbidity (6). Ileus is the absence of peristalsis without mechanical obstruction. Postoperative ileus is delayed return of regular bowel motility after surgery lasting at least 5 days after laparotomy or 3 days after laparoscopy (7, 8). It is associated with abdominal distension, decreased peristaltic sounds, decreased passage of gas, fewer bowel movements, worsening pain, anorexia, nausea and vomiting, and delayed oral intake (5, 8-10). Its other consequences include malnutrition, an increased risk of nosocomial infection, pulmonary complications, deep venous thrombosis, and decreased patient satisfaction (5, 6).

No specific method with which to prevent and treat postoperative ileus has been established (5). Methods in current use are nasogastric suction, early oral intake (11), intravenous fluid administration (10), regional analgesia, decreased intravenous drug use, minimization of intestinal manipulation during surgery, use of cyclooxygenase inhibitors, use of nonsteroidal anti-inflammatory medication, and oral consumption of fluids with a high carbohydrate content (5, 9, 12). Recent studies have suggested that patients chew gum as a strategy to decrease ileus; however, the efficacy of this method was unclear in some studies (13-16) and confirmed in others (17-21).

In an effort to promote maternal and infant health, the World Health Organization has initiated a study of traditional treatments for ileus to discover methods of shortening the length of time to return of bowel motility after caesarean section and decrease the length of hospitalization.

Iranian researchers’ scientific approach to the study of medicine and disease over the centuries makes the study of traditional medical texts a sensible approach to discover new treatments for complicated diseases. 

Avicenna was a great philosopher and an outstanding physician and scientist. His medical text, commonly referred to as *The Canon of Medicine*, was a compendium of medical knowledge of the time based on clinical experience, testing, and observation as well as the historical writings of prominent physicians. Avicenna believed that describing the causes of disease helps to identify different and more successful methods of treatment. Investigations have shown that the use of trials with medicaments recommended by traditional medicine increase the likelihood of discovering new effective treatments (22). Traditional Persian medicine has precisely described the major contributors to ileus and offers a variety of treatments. In traditional medicine, ileus is called *ilavoos*. Physicians of traditional medicine consider ileus to be a form of colic, except that colic usually manifests itself in the colon, while ileus produces small bowel symptoms and is more serious than colic (23-25). Colic and ileus have various causes, but postsurgical ileus appears to originate from gas production in the intestines secondary to loss of sensation in the bowels caused by relaxant and anesthetic drugs (26). As a consequence, the colonlosesitsexcretory power and intestinal paralysis develops (27, 28). The use of remedies that disperse the abdominal gas produced in the digestive tract appears to quicken the return to bowel motility (28, 29).

The transmission of herbal and non-herbal medication through the breast milk to infants of mothers who deliver by caesarean section limit the focus of clinical studies only to those medications that have no negative side effects for the mother or infant. These remedies should also be the most effective for the dispersal of intestinal gas and stimulation of bowel motility. In this respect, *Carum carvi (Bunium persicum Boiss)* was chosen as the most appropriate drug for such studies based on the levels recommended by Iranian books of traditional medicine and permitted PDR (drug information for herbal medicines) (levels for nursing mothers. An oral medication containing *Carum carvi (Bunium persicum Boiss) *was prepared as syrup for evaluation in clinical trials. The aim of study was the effects of Carum carvi (Bunium persicum Boiss) on Early Return of Bowel Motility after Caesarean Section. 

***Hypothesis:*** We hypothesised that in women with caesarean section, Carum carvi (Bunium persicum Boiss) can speed the resumption of postoperative bowel motility.

## Materials and methods

This double-blind, randomized, placebo-controlled trialswas performed from 22 December 2013 to 22 March 2014.

The trial was registered at Iranian Registry of Clinical Trials: (IRCT2013082614486N1).

**Figure 1:**
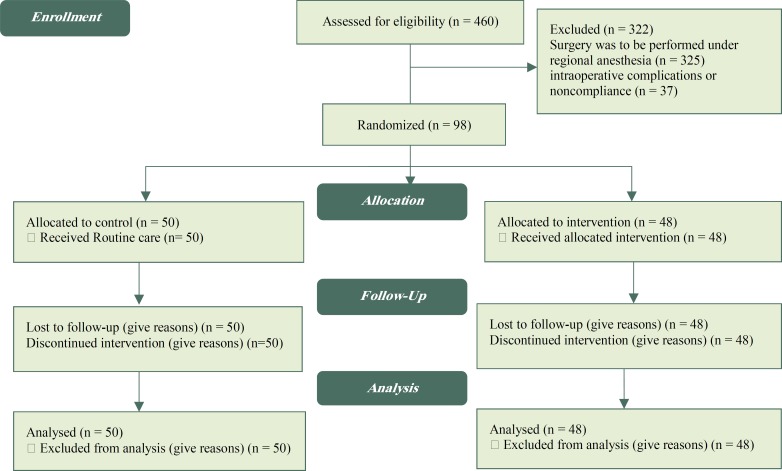
CONSORT 2010 Flow Diagram

98 patients undergoing elective caesarean section under general anesthesia were included in Imam Khomeini Hospital in Sari City, Iran (Figure 1).

Demographic information collected included the patients’ age, gravidity, previous caesarean sections, gestational age, fasting duration, medical and surgical histories, and indications for caesarean section. All operations were elective and carried out in the morning. The operative data collected were the presence of severe adhesions, occurrence of intraoperative complications, estimated blood loss, and duration of surgery. 

The inclusion criteria were having fasted for at least 8 h before surgery, gestational age of 38 to 42 weeks, good vital signs in the mother and infant, no uncommon complications during surgery, and no medical or gynecological disorders such as hypothyroidism, diabetes, or neuromuscular disorders. Patients with postoperative complications such as uterine atony, those requiring more than four doses of antibiotic therapy, those with intraoperative complications (severe adhesions, excessive manipulation of the intestine, massive blood loss or blood transfusion, and injury to bowel or bladder), those with a surgical duration of > 90 min, and those who had received heavy doses of sedatives were excluded.

The syrup consists: 80 gr Carum carvi (Bunium persicum Boiss), 80 gr sugar and 400 ml water. We concentrate the syrup by heating to the wanted consistency thereafter the syrup contains 8 gr Carum carvi (Bunium persicum Boiss) in 20 ml of syrup. We prescribed it to the patients the told dose is 20 ml. The 48 patients in Group A (intervention group) were instructed to slowly drink 10 ml of a syrup containing 8 g of *Carum carvi (Bunium persicum Boiss)* in 20 ml at 6 h postoperatively and another 10 ml at 7 h postoperatively. 

The 50 patients in Group B (control group) were instructed to drink placebo syrup using the same protocol. Both groups received similar routine care after surgery. No patients were given oral or rectal bowel stimulants after caesarean section. The same postoperative rehabilitation program for ambulation was used in both the intervention and control groups. The oral intake of clear fluids and soft foods began when normal bowel sounds were detected and flatus had passed with advancement to a regular diet after the first bowel movement.

All patients were given a nonsteroidal anti-inflammatory medication (diclofenac suppository 100 mg) at 2 and 10 h postoperatively. The need for additional use of anti-inflammatory medication or narcotics was recorded.

Demographic characteristics, time to first hearing of normal intestinal sounds, time to first flatus, time to first bowel movement, and length of hospital stay were compared between the two groups. 

The final sample size was determined according to the results of the pilot study(28). 98 patients undergoing elective caesarean section under general anesthesia.

Women with appointments at the hospital between 22 December 2013 and 22 March 2014 who met the eligibility criteria and agreed to participate were included in the study. Eligible women were randomized to group a (intervention group) and group B (placebo group by random numbers table.

Participants and outcomes assess or were blinded after assignment to interventions.

The time of the end of surgery was designated zero hours. 

In both groups, auscultation for intestinal sounds were performed every 60 min after drinking until the first bowel sounds was heard. Intestinal symptoms such as nausea, abdominal distention, pain, cramping, vomiting, and bowel motility were recorded every 60 min. The patients and their relatives were instructed to record the time to the first passage of flatus, the time to the first bowel movement, and the time until discharge from hospital in the check list provided by the researchers.

Statistical analysis was performed with SPSS version 17 software (SPSS Inc., Chicago, IL). Comparisons between the two groups were performed using the two-tailed Student’s t-test for continuous variables and the chi square or Fisher’s exact test for categorical variables. Throughout all analyses, p < 0.05 was considered statistically significant.

This study was begun after obtaining consent for the methodology from the University of Medical Sciences. Immediately after surgery, patients who fulfilled the inclusion criteria and had provided written consent before the operation were randomly divided into Groups A and B using an odd and even random numbers table. After receiving signed consent forms, a person assigned participants consecutively to the intervention or control arm of the study using a random numbers table. Participants and researcher were blinded.

## Results

During the study period, 460 patients scheduled for elective caesarian section were interviewed; 325 were excluded because surgery was to be performed under regional anesthesia, and 37 were excluded because of intraoperative complications or noncompliance. The demographic characteristics were similar in both groups (Table 1).

Data are expressed as mean ± standard deviation or n (%).

The most common indication for caesarean section was a previous caesarean section (75% vs. 56% in Group A and B, respectively). The two groups showed no significant differences in the indications for primary caesarean section, such as malpresentation and disproportion with refusal of trial of labor or fetal indication. The patients’ intraoperative and postoperative characteristics are shown in Table 2.

**Table 1:** Patients’ demographic and baseline characteristics

	**Group A** **(n = 48)**	**Group B** **(n = 50)**	**P value**
Age (yr)	30.14 ± 5.14	29.72 ± 4.65	0.364
Gestational age	39.04 ± 0.71	38.24 ± 0.95	0.11
Previous abdominal surgery (%)	6.2	8	0.78
Nulliparity (%)	4.1	8	0.036
Fasting duration(hr)	11.62 ± 1.79	10.81 ± 3.06	0.096

**Table 2:** Patients’ intraoperative and postoperative characteristics

	**Group A** **(n = 48)**		**Group B** **(n = 50)**
Duration of surgery(min)	42.29 ± 11.01		45.2 ± 12.81
Postoperative vomiting (%)	3(6.2)		2(4)
Abdominal distension (%)	3(6.2)		10(20)
Postoperative ileus (%)	0(0)		1(2)
Febrile morbidity (%)	4(8.3)		8(16)
Anti-inflammatory medication more than 2 times		2(4.1)	19(38)

Data are expressed as mean ± standard deviation or n (%).

The mean time until detection of the beginning of peristaltic sounds by auscultation, first passage of flatus and first bowel movement as well as the mean hospital stay were significantly different between Groups A and B (Table 3).

The time to recovery of gastrointestinal function was significantly shorter in the study group (Table 3). The first bowel movement occurred within 24 h after caesarian section in 36 (75%) patients in the study group compared with 6 (12%) patients in the control group. The postoperative hospital stay was longer in the control group, but not significantly. One patient in the control group developed severe postoperative ileus; her symptoms resolved with conservative management, including nasogastric tube decompression for 36 h. No adverse events associated with drinking the Carum carvi (Bunium persicum Boiss) syrup occurred during the study.

## Discussion

Compared with the control group, the intervention group had a significantly shorter mean time to hearing the first intestinal sounds, mean time to first flatus, mean time to first bowel movement, and mean length of hospitalization (p < 0.05). No patients developed serious side effects associated with consumption of the syrup.

No previous studies have evaluated the efficacy of *Carum carvi (Bunium persicum Boiss) *or other plants for stimulating intestinal motility after caesarean section. The present study is the first clinical trial to investigate the effectiveness of *Carum carvi (Bunium persicum Boiss) *in reducing the recovery time of bowel motility in women after caesarean section. The findings of the present study were compared with those of other studies that investigated the effect of early oral food intake after caesarean section and the effect of chewing gum on the resumption of bowel motility.

Our data show a beneficial effect of Carum carvi (Bunium persicum Boiss) in terms of shorter mean time intervals to normal intestinal sounds (10.6 vs. 19.5 h), passage of flatus (13.9 vs. 24.8 h), first bowel movement (19.3 vs. 30.7 h), and hospital discharge (31.7 vs. 49.2 h). In the present study, the time intervals to passage of flatus and defecation were generally shorter than those reported with gum chewing or early enteral feeding after caesarean section in previous studies. 

Abd-ul-Mayboud et al. examined the effect of chewing gum on the return of bowel motility and reported that the mean time until first intestinal sounds was 10.9 ± 2.7 and 15.8 ± 3.7 h in the intervention and control groups, respectively. The mean time to the first passage of flatus was 17.9 ± 4.6 and 24.4 ± 7.1 h in the intervention and control groups, respectively. The mean time to the first bowel movement was 21.1 ± 4.7 and and 30.0 ± 8.2 h, respectively, and the mean time to hospital discharge was 40.8 ± 10.6 and 50.5 ± 8.9 h, respectively(14). All patients in their study underwent general anesthesia, similar to the present study. In the present study, the mean difference between the time to the first intestinal sounds in Groups A and B was about 9 h. Because recovery of bowel motility is affected by different factors, this difference appears to be important.

Weinstein et al. administered liquids by mouth 6 h after surgery and heard the first intestinal sounds after 10.3 h in the intervention group and after 14.4 h in the control group (29). These findings are consistent with those of the present study, but the difference between the intervention and control groups was greater in the present study. 

**Table 3:** Primary outcome measures in the study and control groups

	**Group A** **(n = 10)**	**Group B** **(n = 10)**	**P value**
PO intestinal sound heard(hr)	10.66 ± 2.38	19.54 ± 3.85	<0.001
PO passage of flatus(hr)	13.91 ± 3.73	24.82 ± 5.83	0.004
PO passage of motion(hr)	19.31 ± 4.63	30.7 ± 10.21	0.038
PO hospital stay(hr)	31.7 ± 7.75	49.2 ± 10.16	0.05

Data are expressed as mean ± standard deviation.

Adupa et al. recorded a mean time to first intestinal sounds of 32.2 h for the intervention group (early oral intake group) and 24.2 h in the control group (30). Kovavisarach et al. reported that the first intestinal sounds occurred at 28.7 h in the intervention group and 25.5 h in the control group (31). The mean time to first intestinal sounds was higher in these two studies than in other studies. The difference appears to be related to differences in the surgical conditions between countries.

Satji et al. examined the effect of chewing gum after caesarean section in 32 women and reported a 15.5-h difference in the time to the first bowel movement between the two groups (28.4 vs. 43.9 h in the intervention and control groups, respectively) (32).

Teoh et al. reported that the time to the first bowel movement was 44.4 ± 18.7 h in the early oral intake group and 65.6 ± 25.4 h in the control group. The time to hospital discharge was 68.0 h in the intervention group and 69.4 h in the control group. The mean times to first bowel movement, first intestinal sounds, and hospital discharge were significantly different from the results of all previous studies (33). None of the patients developed complications during the study; bloating was reported by four and two patients in the intervention and control groups, respectively.

Important strengths of this study are that loss to follow up was limited.

## Conclusion

The World Health Organization emphasizes the use of traditional medicine, indigenous healing methods, and herbal medicine (34, 35). Additionally, increasingly more doctors and medical students are becoming interested in the use of medicinal plants in treatment, especially in Iran. Therefore, the current study is clinically valuable and necessary.

The data of this study suggest that the use of *Carum carvi (Bunium persicum Boiss) *according to the principles of traditional Persian medicine effectively promotes bowel function after elective caesarean section. The economic impact of early hospital discharge after an uncomplicated caesarean delivery cannot be overlooked, especially in a developing country with limited resources. Further studies using other doses of *Carumcarvi (Bunium persicum Boiss) *and increased intervals between syrup consumption times or other plant in this group as *Carum carvi* L., *Cuminum cyminum* L. are recommended.

 Regarding the effectiveness of drugs components, research about the effect of that principle and vertical components of cumin is need.
